# The enigmatic mechanisms by which *Plasmodium vivax* infects Duffy-negative individuals

**DOI:** 10.1371/journal.ppat.1008258

**Published:** 2020-02-20

**Authors:** Jean Popovici, Camille Roesch, Virginie Rougeron

**Affiliations:** 1 Malaria Molecular Epidemiology Unit, Institut Pasteur du Cambodge, Phnom Penh Cambodia; 2 Malaria Translational Research Unit, Institut Pasteur, Paris & Institut Pasteur du Cambodge, Phnom Penh, Cambodia; 3 Laboratoire MIVEGEC (Université de Montpellier-CNRS-IRD), Montpellier, France; Institut Pasteur, FRANCE

## Abstract

The absence of the Duffy protein at the surface of erythrocytes was considered for decades to confer full protection against *Plasmodium vivax* as this blood group is the receptor for the key parasite ligand *P*. *vivax* Duffy binding protein (PvDBP). However, it is now clear that the parasite is able to break through this protection and induce clinical malaria in Duffy-negative people, although the underlying mechanisms are still not understood. Here, we briefly review the evidence of Duffy-negative infections by *P*. *vivax* and summarize the current hypothesis at the basis of this invasion process. We discuss those in the perspective of malaria-elimination challenges, notably in African countries.

## Author summary

*Plasmodium vivax* is the most widespread of the parasite species causing human malaria. It was considered for decades to be almost absent from sub-Saharan African countries because the majority of people in these areas lack a red blood cell receptor, the Duffy protein, which is the critical gateway for the parasite’s entry into erythrocytes. In the last years, this dogma has been challenged by an increasing number of observations of *P*. *vivax* clinical infections into Duffy-negative people (individuals lacking the Duffy receptor) in many African countries as well as in South America where a significant number of people of African descent are also Duffy-negative. However, we still do not know how *P*. *vivax* is able to infect those individuals. Understanding how this invasion takes place will be critical for the implementation of adapted control solutions targeting this parasite species in order to achieve malaria elimination, especially in African countries where tremendous decline in the prevalence of the other major human malaria species, *P*. *falciparum*, has been achieved in the past decade.

## Introduction

*Plasmodium vivax* is one of the five *Plasmodium* species causing human malaria and although considered as benign during prior decades, it is now recognized as a significant cause of morbidity and mortality in endemic populations [1–4]. Its geographic distribution is the widest, and more than three billion people live within the *P*. *vivax* transmission limits [5]. For decades, this parasite was considered to be almost absent from the African continent with the exception of the Horn of Africa [6, 7]. Indeed, during the era of malariotherapy, it was noted that individuals of African ancestry were often resistant to *P*. *vivax* infection [8–10]. Later on, it was shown that individuals lacking the Duffy antigen, a red blood cell membrane protein known to be absent in several populations of African origin, were protected from *P*. *vivax* infections, establishing the evidence that the receptor of *P*. *vivax* at the erythrocyte surface was the Duffy protein [11–13]. It’s not until the 2000s that the dogma was challenged by reports of Duffy-negative people infected by *P*. *vivax* [14, 15]. Here we review the Duffy nomenclature, the different polymorphisms affecting *P*. *vivax* invasion, the evidence describing infections of Duffy-negative individuals by *P*. *vivax* as well as the hypotheses currently held to explain the molecular basis of Duffy-negative *P*. *vivax* infections. We discuss those in the perspective of malaria-elimination challenges notably in African countries.

## How Duffy affects *P*. *vivax* invasion

The Duffy protein is a receptor for chemokines (Duffy antigen receptor for chemokines [DARC]), officially called the atypical chemokine receptor 1 (ACKR1) [16, 17]. This protein is the receptor for *P*. *vivax* Duffy binding protein (PvDBP), allowing entry of the parasite into the red blood cells and more precisely, into the reticulocytes, the target cells of *P*. *vivax* invasion [18]. Duffy is polymorphic, and many variants have been described, with nomenclature often changing over time. In this Review, we briefly describe the main variants of relevance for interaction with *P*. *vivax*, using the nomenclature commonly used in the field (see [19] for thorough up to date description of the Duffy polymorphism). There are two major alleles known for Duffy: FY*A (coding for the Fy^a^ antigen) [20] and the ancestral allele FY*B (coding for the Fy^b^ antigen) [21–26] (Table 1). Three major genotypes exist (FY*A/FY*A, FY*B/FY*B, and FY*A/FY*B), resulting in three possible phenotypes (corresponding to the associated blood groups): Fy(a+b-), Fy(a-b+), and Fy(a+b+), collectively called Duffy-positive. The Fy(a-b-) phenotype is referred to as the Duffy-negative phenotype [11]. One single point mutation in the GATA-1 transcription factor binding site of the promoter of the gene at position −67 changing a T nucleotide to a C underlies the Duffy-negative phenotype [19, 24]. This genotype is referred to as erythrocyte silent (ES) because individuals homozygous for this mutation lack the Duffy protein on erythrocytes, while heterozygous individuals display approximately 50% of the Duffy protein [27–30]. Duffy-negative people of African ancestry are homozygous for this mutation that is at near fixation in sub-Saharan Africa [31]. In those individuals, the mutation is located upstream of the FY*B allele, leading to the homozygous FY*B^ES^/FY*B^ES^ genotype responsible for the refractoriness of Duffy-negative people to *P*. *vivax* infections.

**Table 1:** Major Duffy alleles, genotypes and phenotypes described in human populations and alternate nomenclature.

	Alleles^a^	Antigen	Genotypes	Phenotypes
Pos	FY*A = FY*01	Fy^a^	FY*A/FY*A	Fy(a+b-)
	FY*B = FY*02	Fy^b^	FY*A/FY*B	Fy(a+b+)
			FY*A/FY*B^ES^	Fy(a+b-)
Neg	FY*A^ES^ = FY*A^null^ = FY*01N.01	Fy^ES^	FY*A/FY*A^ES^	Fy(a+b-)
	FY*B^ES^ = FY*O = FY*B^null^ = FY*02N.01	Fy^ES^	FY*B/FY*B	Fy(a-b+)
			FY*B/FY*B^ES^	Fy(a-b+)
			FY*B^ES^/FY*B^ES^	Fy(a-b-)

^a^The official allele nomenclature is indicated in bold.

Neg, negative, Pos, positive

Discrepant results have been reported concerning the impact on *P*. *vivax* infection of having only one ES allele. It was initially shown in Papua New Guinea that FY*A/FY*A^ES^ heterozygous individuals were at lower risk of *P*. *vivax* infection compared to FY*A/FY*A people (in Papua, the −67 T to C mutation has arisen upstream of the FY*A allele, independently of the FY*B^ES^ found in Africa) [30, 32]. Similarly, in the Brazilian Amazon, heterozygous FY*A/FY*B^ES^ individuals were at lower risk of *P*. *vivax* malaria compared to FY*A/FY*B people [33]. However, in the same Brazilian study, heterozygous FY*B/FY*B^ES^ individuals were at increased risk of malaria infection compared to homozygous FY*A/FY*B people [33]. These apparently conflicting observations might simply reflect differences between the numbers of individuals analyzed for each genotype, resulting in underpowered analysis. More epidemiological studies in different endemic settings are needed to get a clearer understanding of the associations between the Duffy genotypes and infection outcomes.

In addition, independently of the ES genotype, there seems to be a protective effect of the FY*A allele compared to the FY*B one against *P*. *vivax* infections. Indeed, in vitro studies have shown that the PvDBP binding to the FY*A allele was reduced compared to binding to the FY*B allele [34]. It was also observed that FY*A was associated with clinical protection, while FY*B was associated with increased infection risk by *P*. *vivax* [33, 34].

Altogether, those studies suggest that the link between Duffy genotypes and susceptibility to *P*. *vivax* is more complex than just the association between Duffy-negative and full protection.

## Evidence of *P*. *vivax* infections in Duffy-negative people

### Travelers infected by *P*. *vivax* coming back from sub-Saharan African regions

The first indirect evidence of *P*. *vivax* infection in Duffy-negative individuals comes from the reports of travelers presenting a *P*. *vivax* infection after returning from African areas where Duffy-negative is at near fixation [35, 36]. However, those observations did not question the prevailing dogma of Duffy-negative refractoriness for several reasons. First, *P*. *vivax* is morphologically similar to *P*. *ovale* endemic in most Africa, which could lead to microscopic misinterpretation. The advent of molecular techniques to diagnose parasites have ruled out this confounding factor and have allowed to unequivocally identify *P*. *viva*x [37–39]. Second, because of the occurrence of *P*. *vivax* hypnozoites hiding in the liver for months or even years before triggering a relapse infection, the origin of the parasite is difficult to determine. Third, it was suggested that a small proportion of Duffy-positive individuals is enough to sustain the transmission cycle of *P*. *vivax* independently of Duffy-negative infections [40]. Similarly, the detection of *P*. *vivax* in *Anopheles* mosquitoes collected in areas where the majority of people are Duffy-negative cannot conclusively demonstrate Duffy-negative infections [14]. Therefore, additional evidence is clearly needed to demonstrate the ability of *P*. *vivax* to infect Duffy-negative individuals.

### *P*. *vivax* infections in Duffy-negative people from sub-Saharan Africa

In the last decade, reports of *P*. *vivax* infections in Duffy-negative individuals have increased steadily. There is no doubt that the advent of molecular techniques unambiguously detecting *P*. *vivax* at high sensitivity has greatly contributed to those observations. However, those observations come with caveats that can only be resolved by microscopic observations. Indeed, detection of *P*. *vivax* DNA by PCR in a Duffy-negative individual’s blood sample can arise from pre-erythrocytic stages of the parasites even in the absence of actual red blood cell invasion [41]. Similarly, seropositivity to *P*. *vivax* antigens can result from the presence of pre-erythrocytic parasite forms independently of blood-stage infection (not to mention the possible crossreactivity of the serological analysis with other *Plasmodium* species) [42]. Therefore, microscopic observation of *P*. *vivax* within a Duffy-negative erythrocyte, along with genotyping confirmation (especially to exclude a *P*. *ovale* infection), is necessary to prove Duffy-negative infection. The first mention of a microscopic observation of *P*. *vivax* in a Duffy-negative erythrocyte was made in 2006 [14] (Culleton and colleagues [40] mentioned a report by Van Ros in 1985 but we could not retrieve the original publication). However, when the slides were double read by two expert microscopists for confirmation, *P*. *vivax* could not be confirmed [14]. It is in 2010 that the definitive evidence of a *P*. *vivax* infection within Duffy-negative erythrocyte from patients living in Madagascar was reported [15]. It was hypothesized that it is the admixture of Duffy-positive and Duffy-negative people occurring in Madagascar that has allowed the parasites to become adapted to Duffy-negative individuals. Of note, when Bray assessed the susceptibility of 30 Nigerian people to *P*. *vivax* through experimental mosquito bite infections, he did observe *P*. *vivax* blood-stage infection in one of the subjects [43]. It can be speculated that this Nigerian individual was Duffy-negative, though this cannot be confirmed. Interestingly, the strain used by Bray was the “Madagascar strain” of *P*. *vivax*, and it is tempting to speculate that its Malagasy genetic background allowed the parasite to infect a Duffy-negative individual. However, the exact origin of this strain is controversial, and it cannot be ascertained that it indeed comes from Madagascar [2, 44–46]. Since then, further unequivocal *P*. *vivax* Duffy-negative infections were reported from Ethiopia where Duffy-negative and Duffy-positive people coexist [47, 48] and also from countries where Duffy negativity is at near fixation, such as Cameroon [49] and Mali [50] (though in some of those studies the morphological features of erythrocytic *P*. *vivax* were not well resolved). Additionally, the detection of *P*. *vivax* in Duffy-negative individuals either by molecular diagnostic or serological analysis only has been made in a large number of sub-Saharan countries (for extensive review, see [36]). The description of Duffy-negative patients harboring microscopically-confirmed *P*. *vivax* erythrocyte infections coupled to molecular diagnostic confirmation in genotyped Duffy-negative individuals have undoubtedly challenged the established paradigm that prevailed for decades, opening new research questions regarding this neglected malaria parasite.

### *P*. *vivax* infections of Duffy-negative people from the American continent

Current American populations are characterized as an admixture of individuals of different ancestries, due to the historical human migrations over the last centuries (European, African, Native American, Asian, etc.). Depending on the areas, some variable numbers of people are Duffy-negative due to their African ancestry and are distributed throughout South America up to southern United States of America [6]. In South and Central America, where *P*. *vivax* is endemic, the paradigm of Duffy-negative protecting against *P*. *vivax* malaria was quite well established with a number of studies from diverse locations [51–56]. However, similarly to the situation in Africa, several studies have suggested through serological, molecular, and microscopic detection analysis that Duffy negativity is not completely protective against *P*. *vivax* infection [33, 57–59].

All those studies clearly show that the protection of the Duffy negativity does not constitute an absolute barrier against *P*. *vivax* infections.

### Towards understanding the mechanisms of Duffy-negative invasion by *P*. *vivax*

How does *P*. *vivax* invade Duffy-negative red blood cells? Has *P*. *vivax* recently evolved strategies to infect Duffy-negative erythrocytes, or are alternative invasion pathways ubiquitous in *P*. *vivax* populations? Those are key questions, and answering them will be critical to develop strategies to prevent the emergence of Duffy-negative infections. No clear answer has been made yet, mainly because of the inherent difficulty in working on *P*. *vivax* as there is still no in vitro continuous culture available for this species [60, 61]; however, some hypothesis can be raised on the parasite and/or human sides.

### Parasite side: PvDBP duplication and/or other parasite ligands?

The first mechanistic hypothesis that has been made following the observation of Duffy-negative, *P*. *vivax*-infected patients in Madagascar came through whole genome sequencing (WGS) of field isolates. This analysis showed a duplication in the PvDBP gene in Malagasy isolates, and further molecular epidemiology surveys revealed that this duplication was observed in parasites isolated from a wide range of geographic locations (South America, East Africa, Madagascar, Asia-Pacific) [62]. Noteworthy, the highest frequency of isolates with *pvdbp* duplication (nearly 53%) was detected in Madagascar, in comparison to other areas (12.5% in Sudan, 9% in Cambodia). It was thus speculated that this duplication might have been selected in Madagascar to respond to the Duffy negativity barrier perhaps by increasing the amount of PvDBP protein on the merozoite’s surface to bind to an unknown low-affinity receptor [62]. Interestingly, in two different studies, *P*. *vivax*-infecting, Duffy-negative individuals from Ethiopia (where the prevalence of *pvdbp* amplification is between 55% to 80% [63, 64]) carried multiple copies of *pvdbp*, although the number of individuals were too low to draw definitive conclusions (only two in each report) [48, 64]. However, PvDBP itself has been shown to not bind to Duffy-negative erythrocytes, questioning how it could be involved in Duffy-negative invasion [18, 48]. Furthermore, the analysis by WGS of more than 200 *P*. *vivax* isolates from around the world indicated that 33% of Cambodian parasites carried the *pvdbp* duplication, much higher than the previously reported 9% [65]. In fact the initial PCR-based surveys have missed isolates carrying the duplication, because of variation in the boundaries of the duplication among parasites [66]. Using a quantitative PCR assay that enables the gene copy number assessment independently of the duplication boundaries, no difference in the *pvdbp* copy number between Cambodian parasites (where there is virtually no Duffy-negative individuals) and Malagasy ones (where an admixture of Duffy-negative and Duffy-positive individuals coexist) was observed [67]. Those results suggest that *pvdbp* amplification is not selected in response to the Duffy negativity barrier [67]. In a recent molecular epidemiology study conducted on Ethiopian *P*. *vivax* isolates, it was observed that the proportion of parasites with *pvdbp* amplification was higher in individuals carrying the FY*A allele compared to individuals carrying the FY*B one [64]. As mentioned above, because the binding of PvDBP to FY*A is lower than to FY*B, it can be speculated that *pvdbp* amplification could have been selected to increase the affinity to erythrocytes expressing the FY*A allele by supposedly increasing the amount of PvDBP protein at the surface of the merozoites. In the same study, the proportion of parasites with multiple *pvdbp* copies was significantly higher in symptomatic patients compared to asymptomatic individuals, raising the possibility that *pvdbp* amplification is involved in immune evasion of the parasites. Further investigations are clearly needed to understand the role of *pvdbp* amplification.

In addition to copy number variation, there is a number of evidence pointing to the absence of a specific sequence polymorphism in PvDBP in relation to Duffy-negative invasion. Recently, PvDBP sequence analysis of parasites infecting Duffy-negative and Duffy-positive people from Sudan showed that most alleles were shared between parasites, and those alleles were found globally [68]. Also, recombinant PvDBP alleles isolated from two Duffy-negative individuals infected by *P*. *vivax* in Ethiopia were expressed in vitro, and none could bind to Duffy-negative erythrocytes while they were binding to Duffy-positive ones [48].

**Fig 1:**
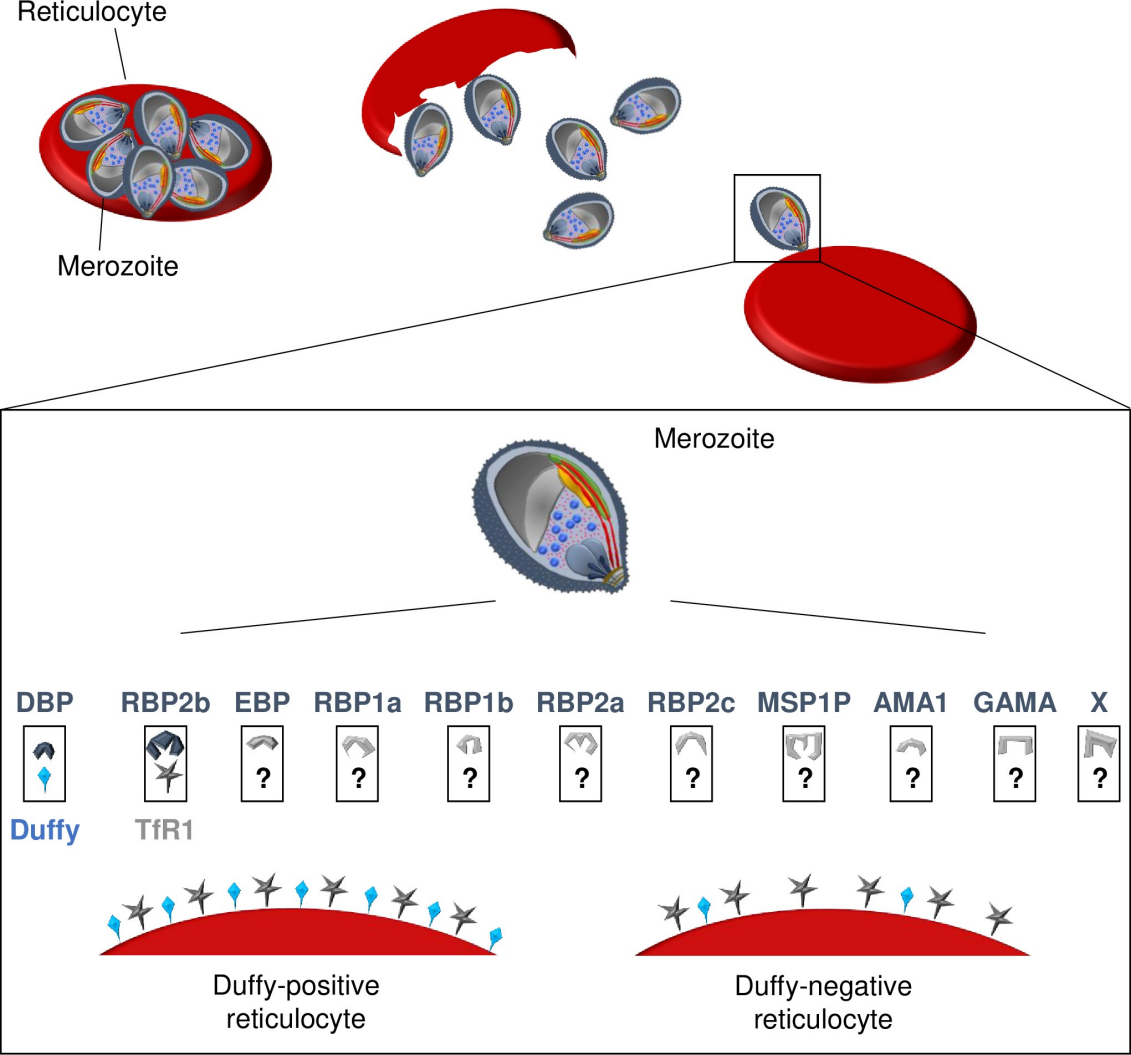
Schematic representation of receptor-ligands involved in *P*. *vivax* invasion process of reticulocytes. At the end of the erythrocytic cycle, the schizonts burst and release merozoites in the blood stream, enabling the invasion of uninfected reticulocytes. The recognition of reticulocytes and the invasion process require interactions between parasite ligands and reticulocyte receptors. For Duffy-positive reticulocyte invasion, PvRBP2b binds first to the TfR1 present on reticulocytes, and subsequently, PvDBP engages with the Duffy protein allowing the entry of the merozoite in the cell. Other ligands such as PvEBP, PvRBPs, PvMSP1P, PvAMA1, or PvGAMA are currently being investigated for their involvement in this invasion process, and their putative receptors are unknown. PvRBP2b probably also recognizes TfR1 of Duffy-negative reticulocytes; however, the subsequent invasion steps are still unknown. Are there a few Duffy molecules present on the surface of the erythrocyte enabling parasites with multiple PvDBP gene copies to invade the cell? Conversely, is the invasion process of Duffy-negative reticulocytes occurring through alternate, yet-to-identity receptors of, perhaps, ligands such as PvEBP, PvMSP1P, or PvGAMA? Finally, the invasion process might occur through complete unknown pathways with both unidentified ligands (noted with X) and receptors. AMA1, anchored micronemal antigen 1; DBP, Duffy binding protein; EBP, erythrocyte-binding protein; GAMA, glycosylphosphatidylinositol-anchored micronemal antigen; MSP1P, merozoite surface protein-1 paralog; Pv, *Plasmodium vivax*; RBP, reticulocyte binding proteins; TfR1, transferrin receptor 1.

There are other parasite ligands described, such as *P*. *vivax* reticulocyte binding proteins (PvRBPs), although they have received much less attention than PvDBP and their role in erythrocyte invasion (whether Duffy-positive or Duffy-negative) for most of them is yet to be determined [69, 70]. PvRBPs are a family of ligands shown to bind to erythrocytes, but conflicting results were obtained for some of them regarding specific binding to reticulocytes [70, 71]. Among the different PvRBPs, the binding to Duffy-negative erythrocytes was evaluated only for PvRBP1a and PvRBP2c, and both were found to bind to Duffy-negative reticulocytes, indicating they could be involved in Duffy-independent invasion pathways [69]. Naturally-acquired antibodies against some RBPs inhibiting binding to erythrocytes or being associated with clinical protection have been described; however, the involvement of those ligands in actual parasite invasion was not assessed, except for PvRBP1a and PvRBP2b [72–77]. While PvRBP1a does not seem to be critical for *P*. *vivax* invasion in Duffy-positive cells [75], PvRBP2b was recently shown to be a key ligand involved in reticulocyte recognition and invasion through the transferrin receptor 1 (TfR1) [76]. However, the roles of PvRBP1a and of PvRBP2b–TfR1 have not been evaluated specifically in the invasion of Duffy-negative red blood cells. Recently, *P*. *vivax* merozoite surface protein-1 paralog (PvMSP1P) and *P*. *vivax* glycosylphosphatidylinositol-anchored micronemal antigen (PvGAMA) were shown to bind to both Duffy-positive and Duffy-negative red blood cells, and antibodies against PvMSP1P blocked the related *P*. *knowlesi* invasion in Duffy-positive reticulocytes [78–80]. Those results suggest that PvGAMA and PvMSP1P are ligands that could be involved in Duffy-independent reticulocyte invasion pathways, although functional demonstration is still lacking. Finally, *P*. *vivax* erythrocyte-binding protein (PvEBP, also known as PvDBP2) has also been shown to moderately bind to Duffy-negative reticulocytes [48, 81, 82]. Of interest, it was recently shown that this gene can be amplified and a higher proportion of parasites from Madagascar (where Duffy-negative and Duffy-positive people coexist) carry multiple copies of PvEBP compared to isolates from Cambodia (where only Duffy-positive people are present) [67]. Also, while only duplication was observed in Cambodia, up to five copies of PvEBP were detected in Malagasy parasites [67]. Altogether, those results suggest that PvEBP might be also involved in Duffy-negative invasion mechanisms, but again, functional demonstration is still lacking. Functional studies need to be developed in the future to figure out the role of each of the mentioned parasite ligands in the invasion of Duffy-positive and Duffy-negative reticulocytes as well as to identify possible unknown ligands involved in alternative invasion pathways (Fig 1).

### Human side: Alternate receptor or Duffy involvement?

On the human erythrocyte side, the main question is: Through what receptor is *P*. *vivax* able to enter Duffy-negative red blood cells? While for *P*. *falciparum* many receptors have been described to ensure the parasite entry through multiple pathways, for *P*. *vivax* (until recently) only the Duffy protein was known. As mentioned above, TfR1 has recently been identified as the receptor to PvRBP2b for reticulocyte recognition and invasion [76]. TfR1 is one of the many membrane proteins lost during red blood cell maturation and is thus absent from normocytes [83, 84]. It is present on both Duffy-positive and Duffy-negative reticulocytes; however, it is believed that the critical interaction between PvRBP2b and TfR1 occurs upstream of the PvDBP–Duffy one, not independently of it [76]. Anyway, the role of this interaction in Duffy-negative has not been assessed, and an alternate human red blood cell receptor involved in Duffy-independent invasion pathways has yet to be discovered.

Could the invasion of Duffy-negative people require a particular Duffy protein not recognized by serological tools and therefore falsely assigned as negative? Probably not, as sequencing of this gene has consistently shown that the basis for Duffy-negative is a single mutation in the promoter of the gene upstream of the FY*B allele in African populations [24]. More conclusively, the full coding sequence of the Duffy gene, including its promoter, of 14 Duffy-negative individuals actually infected by *P*. *vivax*, have been sequenced, and all were homozygous with the expected −67 T to C mutation upstream of the FY*B alleles, indicating that no cryptic allele was at the origin of the infection [15].

However, what has not been definitely ruled out and is perhaps the most parsimonious explanation for the mechanisms of Duffy-negative invasion is that the Duffy-negative phenotype is not Duffy null [85]. Indeed, it can be speculated that although the −67 T to C mutation reduces the binding of the GATA-1 transcription factor and therefore transcription of the gene, this reduction might not be complete and RNA transcription could still be occurring at very low levels, leading to an amount of Duffy protein below the limit of detection of current analytical tools. Some recent data suggest that Duffy protein is notably detectable in erythroid precursor cells typically found in the bone marrow where *P*. *vivax* invasion is believed to occur to some extent [85–87]. It is worth mentioning here that Duffy-negative people do express the Duffy protein in other cell types such as in endothelial cells, indicating that the expression of Duffy is not intrinsically abolished by the −67 T to C mutation [23, 88]. This hypothesis would explain the very low parasitemia usually observed in Duffy-negative infected patients and also imply that *P*. *vivax* does not necessarily require an alternate pathway but could instead go through the classical PvDBP−Duffy invasion process. Such a hypothesis is so far only a speculation, and the involvement of an unknown receptor cannot be excluded (Fig 1). More investigations are needed to determine how the parasite is able to infect Duffy-negative red blood cells.

## Future directions and conclusive remarks

With such evidence of *P*. *vivax* infections in Duffy-negative people, a better understanding of the epidemiology of this parasite is necessary and will involve the implementation of specific diagnostics in the African continent, where apart from the Horn of Africa and Madagascar, *P*. *vivax* is usually not looked for. A characteristic of *P*. *vivax* infections in Duffy-negative individuals is the usually low parasitemia easily missed by microscopy or conventional rapid diagnostics tests (RDTs) and leading to asymptomatic infections often detected through community-based, cross-sectional surveys [50, 89]. As such, sensitive molecular detection tools such as PCR should be implemented for *P*. *vivax* diagnostics.

To provide better insights in the mechanisms underlying Duffy-negative infections, next-generation sequencing technologies will be extremely useful both at the genomic and transcriptomic level. By identifying genomic loci under selection in *P*. *vivax* isolates infecting Duffy-negative individuals compared to Duffy-positive ones or originating from areas where Duffy negativity is at near fixation compared to areas where Duffy-positive are predominant, candidate genes involved in the adaptation to Duffy-negative reticulocytes might be detected. Similarly, by comparing the gene expression profiles of parasites infecting Duffy-negative or Duffy-positive people, the signature of genes specifically regulated to respond to the Duffy negativity barrier might be identified. Such an approach could also make use of the *Saimiri* and *Aotus* nonhuman primate model—as both species can be infected by *P*. *vivax*, while PvDBP can bind only to *Aotus* erythrocytes and not to *Saimiri* ones, indicating an alternative invasion pathway reminiscent of what is observed for Duffy-negative human erythrocytes [90]. Alternative models to decipher erythrocyte invasion mechanisms could also rely on the genetically tractable *P*. *knowlesi* that shares many biological similarities with *P*. *vivax*, including the requirement of the Duffy receptor for erythrocyte invasion [91] or even on *P*. *cynomolgi*, closely related to *P*. *vivax* and recently adapted to in vitro culture [92].

Providing definitive evidence of the involvement of any candidate ligand involved in Duffy-negative reticulocytes’ invasion will anyway require functional demonstration in a *P*. *vivax* human reticulocyte model. However, as mentioned before, there is still no continuous in vitro culture for this parasite species, and consequently no genetic transformation is possible. Only tedious, short-term cultures conducted in a handful of laboratories around the world can be performed [76, 93–95]. Nevertheless, using those assays in order to evaluate the capacity of parasites with different genotypes (i.e., single or multicopy *pvdbp* parasites) to invade Duffy-negative reticulocytes in presence or absence of monoclonal antibodies specific for a candidate ligand or receptor will allow one to determine the pathways used by *P*. *vivax* to infect Duffy-negative erythrocytes. In addition, on the erythrocyte side, elegant technologies have been developed to manipulate the repertoire of proteins expressed on erythropoietic cells using an immortalized erythroid progenitor cell line that can provide additional evidence on the involvement of specific receptors in *P*. *vivax* invasion [96]. All those approaches, combined with structural biology techniques, have the potential to identify receptor–ligand interactions involved in *P*. *vivax* invasion as performed for the recent discovery of PvRBP2b–TfR1 involvement [76].

In Africa, malaria burden is largely due to the more virulent and severe *P*. *falciparum* that is, of course, currently the primary target of elimination strategies. However, it can be expected that in the future while the elimination of falciparum malaria hopefully continues as seen for the last decade [97], more cases of *P*. *vivax* will be reported, as often the proportion of *P*. *vivax* cases increases while elimination progresses [5]. In those countries and elsewhere, in order to achieve elimination of all malaria species it will be necessary to implement strategies targeting *P*. *vivax* [98]. Among those, a PvDBP-based vaccine is currently under clinical development [99, 100], and deciphering the molecular pathways by which *P*. *vivax* is able to infect Duffy-negative people will be critical for ensuring the success of such a strategy.
